# Ecosystem Management and Ecological Modeling

**DOI:** 10.1100/tsw.2002.80

**Published:** 2002-01-14

**Authors:** S.E. JØrgensen

**Affiliations:** DFH, Institute A, Environmental Chemistry, University Park 2, 2100 Copenhagen Ø, Denmark

**Keywords:** ecosystem management, ecological engineering, ecological modeling, structurally dynamic models

## Abstract

It is the intention of this paper to demonstrate that environmental technology must be supplemented by other tools to be able to solve environmental problems properly. Five cases are used to illustrate the possibilities of ecological engineering, a new engineering field based on ecology, as chemical engineering is based on chemistry. It encompasses restoration of ecosystems, utilization of ecosystems to the benefit of both mankind and nature, construction of ecosystems, and ecologically sound planning of ecosystems from a holistic point of view. Ecological engineering requires a good knowledge of the system properties of ecosystems to be able to fully utilize the possibilities that ecosystem management offers. Models reflecting the ecosystem properties are furthermore needed to be able to quantify the effects of the ecological engineering solutions to the environmental problems. This is clearly demonstrated in two of the five case studies presented in the paper.

## INTRODUCTION

Billions of dollars have been invested in environmental technology during the last couple of decades in the industrialized countries, and the pollution problems are still far from being solved. Advanced wastewater treatment has, for instance, been used to solve the eutrophication problems of many lakes in Japan, North America, and Europe — and in the best cases the eutrophication has stabilized or has only been reduced slightly. It has been increasingly clear during this period that environmental technology does not suffice.

In addition to environmental technology, it is necessary to manage ecosystems more effectively and to consider all types of ecological impacts in order to be able to solve the problems properly. For a lake, for instance, it will be necessary to consider all activities in the entire drainage area, as all activities may directly or indirectly influence the water quality of the lake.

A new engineering discipline has been developed as a result of this experience: ecological engineering. It covers restoration of ecosystems, ecologically sound management of ecosystems, utilization of ecosystems to solve pollution problems, and construction of artificial ecosystems. A subdiscipline to ecotechnology or ecological engineering, named ecohydrology, has also been proposed as a tool to solve important environmental problems associated with aquatic ecosystems. The ideas behind ecohydrology are that the hydrology of aquatic ecosystems has a major influence on the water quality and the entire ecosystem, and that the biological components of the ecosystem influence the hydrology — for instance a channel with a dense vegetation will obviously have a different hydrology than a channel without vegetation.

Ecosystem management, which is defined as ecological engineering, including ecohydrology, already has a wide and growing application, because it is a cost-effective supplement to environmental technology. The observed effects are often surprisingly good, compared with the costs. It is, however, clear from past applications that a quantitative relationship between the measures taken and the effects expected is urgently needed to give the best result from the management actions. This implies that ecosystem models should be developed for many ecological engineering applications and that ecological modeling is one of the most important tools for the ecological engineer.

Ecological models have been in use as a management tool for at least the last 3 decades. An ecological model is developed to simulate the important processes relating the state variables (important ecological attributes describing the state of the ecosystem: for instance the water quality) to forcing functions (the impacts on the ecosystems). Ecological models have been used to predict the water quality in the future by assumption of a specific development — for instance, in the population growth of a drainage area, to determine the self-purification ability of an ecosystem of interest, or its assimilative capacity. Lately, an increasing number of models have been developed to assess the value of ecological indicators, to get an indication of the sustainability of the environmental management strategy, or as a tool in ecological engineering. To give the full use of these models, this paper will hopefully encourage more ecologists, ecological engineers, and environmental managers to publish their models as papers to the benefit of one another.

To illustrate the message of this paper — "We can manage the environment better by using ecosystem management, and we manage ecosystems better by using ecological models" — two models developed to serve as tools for ecosystem management and their initial applications are presented below. A few possible applications of ecological models in ecosystem management are also discussed. The experience gained by these case studies will be discussed to support the message. A short introduction is given in the next section to describe the state-of-the-art biogeochemical dynamic models that are used effectively today in ecological engineering, including ecohydrology

## STRUCTURALLY DYNAMIC BIOGEOCHEMICAL MODELS

Models can be considered a synthesis of our knowledge, whether it is observations or general knowledge about the system or the state variables of the model[1]. It is a shortcoming of many models that they do not account for the adaptation of the organisms to stresses or the shifts in the species composition, which we know take place in the ecosystems. However, a new type of model, named structurally dynamic model (SDM), uses either expert knowledge or a goal function to describe these processes resulting in a change in the parameters of the model. Thirteen models have used exergy, which measures the distance from thermodynamic equilibrium[2,3,4,5,6,7], as a goal function to describe the change of the parameters when the forcing functions have been changed. By use of relationships for most of the parameters of phytoplankton and zooplankton and the sizes of these organisms, the optimization procedure can be facilitated significantly[8]. There are also good examples of SDM based upon expert knowledge; see, for instance, Recknagel et al.[9], Reynolds[10], and Patten[11].

Adaptation and shifts in species composition are associated with properties that are better fitted for survival under the currently changed conditions. Survival implies maintenance of the biomass, and growth means increase of biomass. It costs exergy to construct biomass, and biomass therefore possesses exergy, which is transferable to support other exergy (energy) requiring processes. Survival and growth can therefore be measured by use of the thermodynamic concept of exergy.

Darwin's theory, therefore, could maybe be reformulated in thermodynamic terms as follows: the prevailing conditions of an ecosystem steadily change, and the system will continuously select the species, and thereby the processes, that can contribute most to the maintenance or even growth of the exergy of the system.

Ecosystems are open systems and receive an inflow of solar energy. It carries low entropy, while the radiation from the ecosystem carries high entropy.

Notice that the thermodynamic translation of Darwin's theory requires that populations have the properties of reproduction, inheritance, and variation. The selection of the species that contribute most to the exergy of the system under the prevailing conditions requires that there are enough individuals with different properties so that a selection can take place — it means that the reproduction and the variation must be high and that, once a change has taken place due to better fitness, it can be conveyed to the next generation.

Notice also that the change in exergy is not necessarily $\geq 0$; it depends on the changes of the resources of the ecosystem. The proposition claims, however, that the ecosystem attempts to reach the highest exergy level that is possible, under the given circumstances, and with the genetic pool available and ready for this attempt[12,13].

It is not possible to measure exergy directly — but it is possible to compute it — if the composition of the ecosystem is known. Jørgensen and Mejer[13] have shown, by the use of thermodynamics, that the following equation is valid for the components of an ecosystem:

$$Ex = RT \sum_{i=1}^{i=n} (C_i * ln (C_i / C_{eq,i}) - (C_i - C_{eq,i})) \qquad (1)$$

where R is the gas constant; T is the temperature of the environment (Kelvin); $C_i$ represents the $i^{th}$ component, expressed in a suitable unit (e.g., for phytoplankton in a lake, $C_i$ could be milligrams of a focal nutrient in the phytoplankton per liter of lake water); $C_{eq,i}$ is the concentration of the $i^{th}$ component at thermodynamic equilibrium, which can be found in Morowitz[14]; and n is the number of components. $C_{eq,i}$ is, of course, a very small concentration of organic components, corresponding to the probability of forming a complex organic compound in an inorganic soup (at thermodynamic equilibrium). Morowitz[14] has calculated this probability and found that for proteins, carbohydrates, and fats the concentration is about $10^{-86}$ μg/l, which may be used as the concentration at thermodynamic equilibrium.

The idea of the new generation of models presented here is to find continuously a new set of parameters (limited for practical reasons to the most crucial, i.e., sensitive parameters) that is better fitted for the prevailing conditions of the ecosystem. "Fitted" is defined in the Darwinian sense by the ability of the species to survive and grow, which may be measured by the use of exergy (see Jørgensen[2,3,6,15,16,17] and Jørgensen and Mejer[12]). Fig. 1 shows the proposed modeling procedure, which has been applied in some of the cases presented below.

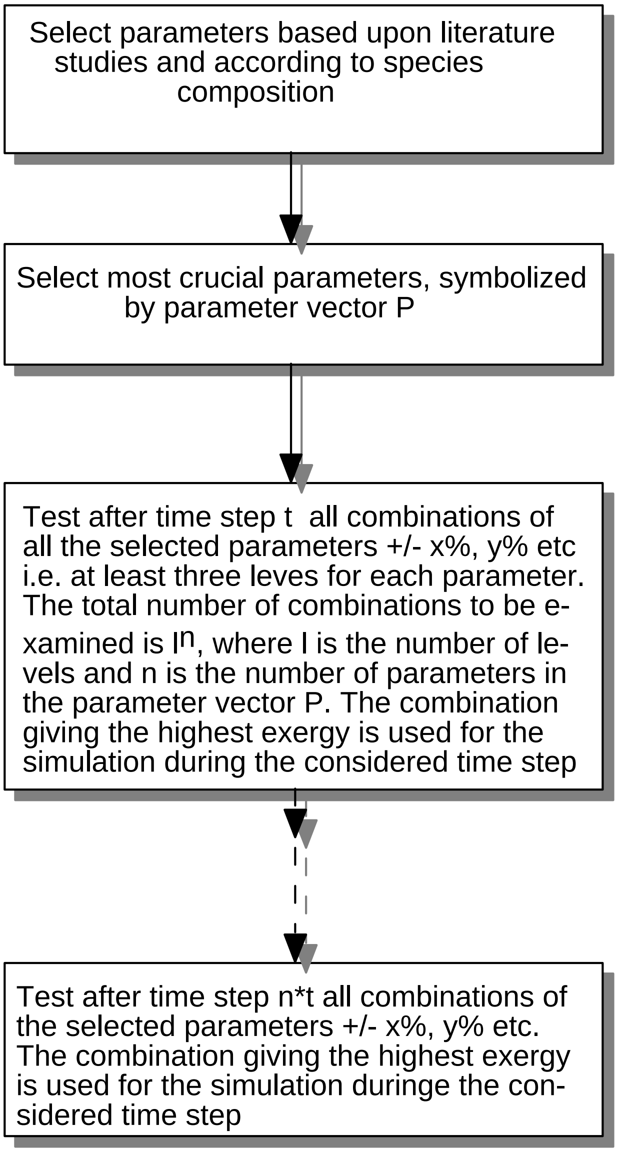

**FIGURE 1.** Procedure applied for development of SDM.

An ecologically useful exergy index can be computed, based on concentrations of chemical components, $c_i$, multiplied by weighting factors, $ß_i$, reflecting the exergy contents of the various components due to their chemical energy and the information embodied in DNA:

$$Ex = \sum_{i=0}^{n} ß_i \, c_i \qquad (2)$$

Values for $ß_i$, based on detritus exergy equivalents, are available for a number of different species and taxonomic groups. The unit, detritus exergy equivalents, expressed in g/l, can be converted to kJ/l by multiplication by 18.7, which corresponds approximately to the average energy content of 1 g detritus[14]. The index, i = 0 for constituents, covers inorganic components, but in most cases these will be neglected, as contributions from detritus and living biota are much higher, due to extremely low concentrations of these components in the reference system. Our exergy index

**TABLE 1**
**Approximate Numbers of Nonrepetitive Genes**

| Number of Organisms | Information Genes | Conversion Factor[a] |
|---|---|---|
| Detritus (reference) | 0 | 1 |
| Minimal cells | 470 | 2.7 |
| Bacteria | 600 | 3.0 |
| Algae | 850 | 3.9 |
| Yeast | 2000 | 6.4 |
| Fungi | 3000 | 10.2 |
| Sponges | 9000 | 30 |
| Molds | 9500 | 32 |
| Trees | 10,000 – 30,000 | 30 – 87 |
| Jellyfish | 10000 | 30 |
| Worms | 10,500 | 35 |
| Insects | 10,000 – 15,000 | 30 – 46 |
| Zooplankton | 10,000 – 15,000 | 30 – 46 |
| Fishes | 100,000 – 120,000 | 300 – 370 |
| Amphibians | 120,000 | 370 |
| Birds | 120,000 | 390 |
| Reptiles | 130,000 | 400 |
| Mammals | 140,000 | 430 |
| Humans | 250,000 | 740 |

[a] Based on numbers of *information genes* and the *exergy* content of organic matter in the various organisms, compared with the exergy content of detritus (about 18 kJ/g). For further detail, see Jørgensen[4].

therefore accounts for the chemical energy in organic matter plus the information embodied in living organisms. The information is contained in the right amino acid sequence of proteins, which explains the extreme low concentration at thermodynamic equilibrium. It is measured from the extremely small probabilities of forming living components spontaneously from inorganic matter. The weighting factors, $ß_i$ (see Table 1), where rough estimations are given, may be considered as quality factors reflecting the extent to which different taxa contribute to overall exergy. The exergy index calculated by Eq. (2) has been used successfully as goal function to develop structurally dynamic models according to the procedure in Fig. 1. For further detail on exergy and how Eq. (2) can be shown to be a reasonable approximation see Jørgensen[4,5], Jørgensen et al.[7], and Jørgensen and de Bernardi[18].

## ECOHYDROLOGICAL SOLUTION TO EUTROPHICATION OF RESERVOIRS

A number of reservoirs are exposed to eutrophication. This case illustrates how eutrophication can be reduced by the use of ecohydrology, which should be considered in addition to the use of environmental technology. The developed model presumes that phosphorus is the limiting factor in a reservoir and therefore describes only the phosphorus cycle. It has eleven state variables, namely, dissolved reactive phosphorus, phosphorus in phytoplankton, phosphorus in zooplankton, phosphorus in planktivorous fish, phosphorus in their eggs, phosphorus in carnivorous fish, phosphorus in their eggs, phosphorus in detritus, exchangeable phosphorus in the sediment, phosphorus in the pore water, and the lake volume. The conceptual diagram of the model is shown Fig. 2. The equations are not very different from other eutrophication models, which means that the Michaelis-Menten expression is used for phytoplankton growth and zooplankton grazing, while mineralization follows a first-order reaction. Only phosphorus is considered as a

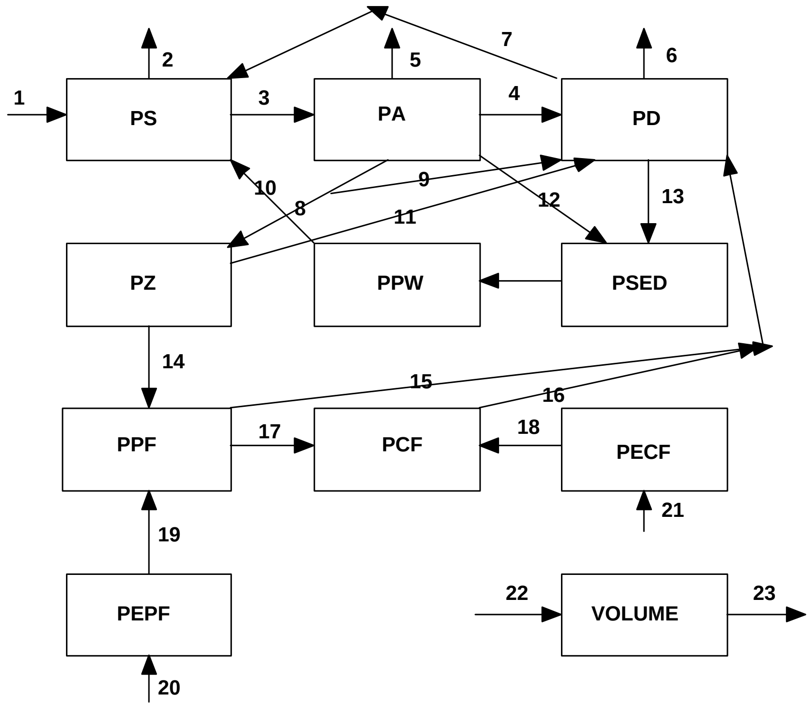

**FIGURE 2.** The conceptual diagram. The model has 11 state variables: PS is the dissolved P; PA, the P in phytoplankton; PZ, the P in zooplankton; PPF, P in planktivorous fish; PCF is P in carnivorous fish; PD, P in detritus; PSED, P in sediment; PEPF, P in eggs of planktivorous fish; PECF, P in carnivorous fish; PPW, P in pore water in the sediment; and volume is the lake volume. The processes are (only P is considered): (1) inflow of P, (2) outflow of P, (3) uptake of P by phytoplankton, (4) mortality of phytoplankton, (5) outflow of phytoplankton, (6) outflow of detritus-P, (7) mineralisation, (8) grazing by zooplankton, (9) faeces originated from grazing, (10) diffusion of pore water phosphorus, (11) mortality of zooplankton, (12) settling of phytoplankton-P, (13) settling of detritus-P, (14) predation on zooplankton by planktivorous fish, (15) mortality of planktivorous fish, includes the faeces originated from the predation, (16) mortality of carnivorous fish, included faeces from process 17, (17) predation on planktivorous fish by carnivorous fish, (18) recruitment from eggs of carnivorous fish, (19) recruitment from eggs of planktivorous fish, (20) production of planktivorous fish eggs, (21) production of carnivorous fish eggs, (22) inflow of water, (23) outflow of water. Notice that the processes 18 and 19 are highly dependent on the water level.

limiting element. Fig. 3 shows a simulation result for the four important biological components, phytoplankton-P, zooplankton-P, planktivorous fish-P and carnivorous fish-P.

The amount of water flowing into the reservoir is covered by a forcing function, giving the water flow rate as the inverse residence time, which varies between 0.005 and 0.05, corresponding to a residence time between 20 and 200 days. The key question to answer with the model is: how does the flow rate out of the reservoir influence the biological factors? The model was therefore used to simulated two scenarios: one corresponding to a constant outflow rate, and one corresponding to a higher outflow rate when the planktivorous fish are spawning and the phytoplankton is blooming, because that would imply faster washout of the eggs and phytoplankton. The residence time will, of course, also have an effect on the water depth. At lower water depth, the fish will spawn on more sandy sediment without vegetation, and at higher water level the fish will spawn on sediment covered by vegetation. The mortality of the eggs is much higher on the former sediment than on the later one. The influence of the water depth on the survival of the eggs is therefore included in the model.

The model is used to set up two scenarios: one with a constant water outflow rate, and one with a higher outflow rate in the period of April 1 to August 1, giving a higher washout rate and

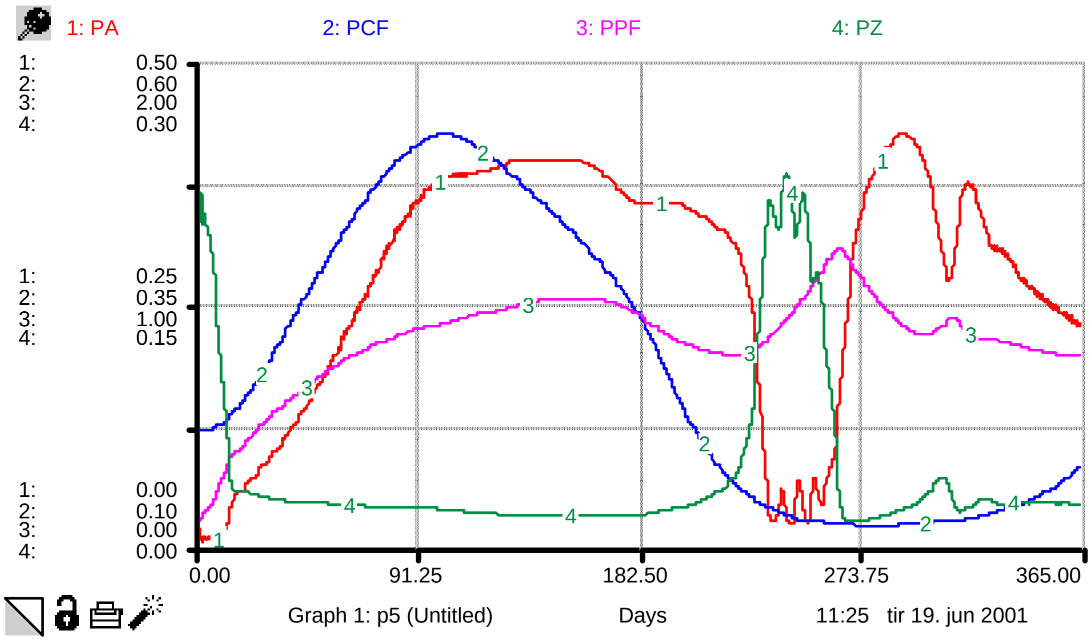

**FIGURE 3.** The simulation results for P in phytoplankton (PA), P in zooplankton (PZ), P in planktivorous fish (PPF), and P in carnivorous fish (PCF) are shown for a year in the case that P in the in-flowing water is 0.5 mg/l.

lower water level. The two scenarios have been determined at four phosphorus concentrations, namely 0.05 mg/l (oligotrophic), 0.2 mg/l (mesotrophic), 0.5 mg/l (eutrophic), and 1.0 mg/l (almost hypereutrophic).

The model is very general and has not been calibrated or validated, as it is not referring to any specific case study. The quantitative results can therefore not be used for a specific case study, but the results should be interpreted as a typical pattern of the reactions of biological components to changes in the hydrological forcing functions. The results can therefore only be used to demonstrate ecohydrological possibilities of an improved reservoir management. In real cases, a specific model should be developed and the model must be calibrated and validated using observations from the reservoir.

It is expected that a higher flow rate during the period of April 1 to August 1 (with a lower flow rate during the rest of the year) implies a faster washout of the planktivorous eggs and of phytoplankton and a higher mortality of the fish eggs, due to the lower water level. The application of the structurally dynamic approach implies that the growth rate of the zooplankton decreases (which means that the size is increasing) as the phosphorus concentration decreases, which is in accordance with the general observations (see, for instance, Jørgensen and de Bernardi[18] on the application of biomanipulation). The total effect will therefore be a decrease in phytoplankton and fish, particularly planktivorous fish. The effect on zooplankton is difficult to predict, because the predator and the food decrease at the same time. Because the entire ecosystem is working as an interrelated network, it is hardly possible to quantify the consequences of changed hydrology on the biological components without a model. The results of applying the presented model are summarized in Table 2. It is possible to conclude, not surprisingly, that the hydrology has a major impact on the biological components. It is possible to reduce eutrophication and the planktivorous fish significantly by changing the flow regime, which is completely in accordance with Zalewski and Wagner[19].

**TABLE 2**
**Quantification of Ecohydrological Effect on Biological Components**

| Biological Component | P in-flow (mg/l) | Spring and Summer Peaks (mg P/l) | | | | |
|---|---|---|---|---|---|---|
| | | Constant Outflow Rate | | High Outflow Rate 1/4-1/8 | | Difference |
| Phytoplankton | 1.0 | 0.52 | 0.46 | 0.49 | 0.041 | down 8% |
| | 0.5 | 0.27 | 0.19 | 0.18 | 0.15 | down 28% |
| | 0.2 | 0.046 | 0.037 | 0.044 | 0.032 | down 8% |
| | 0.05 | 0.042 | 0.028 | 0.040 | 0.025 | down 7% |
| Zooplankton | 1.0 | 0.045 | 0.18 | 0.17 | 0.19 | up 37.5% |
| | 0.5 | 0.075 | 0.085 | 0.07 | 0.1 | up 6% |
| | 0.2 | 0.26 | 0.05 | 0.25 | 0.15 | up 29.0% |
| | 0.05 | 0.15 | 0.04 | 0.17 | 0.06 | up 21.1% |
| Planktivorous fish | 1.0 | 0.95 | 0.87 | 0.95 | 0.82 | down 2.8% |
| | 0.5 | 0.85 | 0.62 | 0.70 | 0.56 | down 14.3% |
| | 0.2 | 0.27 | 0.18 | 0.26 | 0.14 | down 11.1% |
| | 0.05 | 0.25 | 0.14 | 0.24 | 0.10 | down 12.8% |
| Carnivorous fish | 1.0 | 0.48 | | 0.49 | | up 2.1% |
| | 0.5 | 0.46 | | 0.47 | | up 2.2% |
| | 0.2 | 0.40 | | 0.43 | | up 7.5% |
| | 0.05 | 0.34 | | 0.35 | | up 2.9% |

## MODELING THE USE OF PHYTOREMEDIATION

The numbers of registered contaminated sites are alarming and continue to grow. Environmental remediation is thus an admittedly rapid growth area, ripe for technological application and innovation.

The high cost of remediation has driven the interest in the direction of ecological engineering, such as applications of bioremediation technologies. Phytoremediation is an attractive alternative to clean up polluted sites, because it is often possible to solve the pollution problem satisfactorily by this method, without the hazard and expense involved in removing polluted materials for treatment elsewhere, using traditional environmental technological methods.

Phytoremediation may be applied to both organic waste and heavy metal contaminations, although the methods used may differ. A practical example of heavy metal removal by phytoremediation is therefore discussed below.

The success of any phytoremediation technology depends on a number of factors, including site characteristics and environmental factors such as temperature, pH, redox potential, concentrations of nutrients, the contaminant, the presence of microorganisms, the choice of plants, and bioavailability. All these factors work simultaneously and influence each other. Therefore, it is absolutely necessary to use a model to be able to predict the result of phytoremediation. A model for the uptake of heavy metals by plants has therefore been developed as a management tool in the application of phytoremediation.

Plants are contaminated by heavy metals originating from deposition of heavy metals (waste sites), air pollution, the application of sludge from municipal wastewater plant as a soil conditioner, from contaminated soil, and from the use of fertilizers.

The uptake of heavy metals by plants has previously been modeled[20]. The model can briefly be described as follows. Depending on the soil composition (pH, content of clay and humus, and the ion exchange capacity, CEC [cation exchange capacity]) it is possible to find, for various heavy metal ions, a distribution coefficient, i.e., the fraction of the heavy metal that is dissolved in the soil water relative to the total amount. The distribution coefficient was found by examination of the dissolved heavy metals relative to the total amount for several different types of soil. Correlation between pH, the concentration of humic substances, clay and sand in the soil on the one hand, and the distribution coefficient on the other, was also determined. The uptake of heavy metals was considered a first-order reaction of the dissolved heavy metal.

It is also possible to use acid-volatile sulfide and organic carbon to describe the metal binding capacity of sediment in constructed wetlands. This will give approximately the same ratio of “bound” to “bioavailable” heavy metals as the above-mentioned correlation. The basic idea is the same, namely to find easily measurable soil properties that determine the metal binding capacity, which is crucial for the uptake of heavy metals by plants.

In addition, to the uptake from soil water, the model presented below considers:

1. The direct uptake from atmospheric fallout onto the plants,
2. The other sources of contamination, such as fertilizers, and
3. The long-term release of heavy metal bound to the soil and the unharvested parts of the plants.

Published data on lead and cadmium contamination in agriculture are used to calibrate and validate the model, which is intended to be used for:

1. A more generally applicable risk assessment for the use of fertilizers and sludge that contain heavy metals as contaminants,
2. A risk assessment for the use of plants harvested from a waste site, and
3. Determining the possibilities of removal of heavy metals by plants that have a particular ability to take up heavy metals.

This last intended application of the model makes it useful for determination of the result of application of phytoremediation.

Fig. 4 shows a conceptual diagram of the Cd-version of the model. The STELLA software was applied. As can be seen, it has four state variables: Cd-total, Cd-soil, Cd-detritus, and Cd-plant. An attempt was made to use only one or two state variables for cadmium in the soil, but to get an acceptable level of agreement between data and model output, three state variables were needed. This can be explained by the presence of several soil components that bind the heavy metal differently; see Christensen[21,22], EPA, Denmark[23], Hansen and Tjell[24], Jensen and Tjell[25], and Chubin and Street[26]. Cd-total covers the cadmium bound to minerals and to more-or-less refractory material; Cd-soil covers the cadmium bound by adsorption and ion exchange; while Cd-detritus is the cadmium bound to organic material with a wide range of biodegradability.

The forcing functions are: Cd from air pollution, Cd from sludge and fertilizers, yield, and loss. Contaminated soil will obviously have a high concentration of cadmium in the Cd-total and Cd-soil compartments as initial values.

The atmospheric fallout is known, and so is the allocation of this source to the soil and to the plants (Processes 2, 3, and 4; see Fig. 4)[24,25]. Cd-input (Process 1; see Fig. 4) covers the heavy metal in the fertilizer, sludge, compost, and other added external sources. The yield corresponds to the part of the plants that is harvested (Process 8). This is a pulse function at day 180, with an occurrence, afterward, every 360 days. It is, in this case, considered to be 40% of the plant biomass.

The loss covers transfer to the soil and groundwater below the root zone. It is expressed as a first-order reaction, with a rate coefficient dependent on the distribution coefficient that is found from the soil composition and pH, according to the correlation found by Jørgensen[20]. Furthermore, the rate constant is dependent on the hydraulic conductivity of the soil.

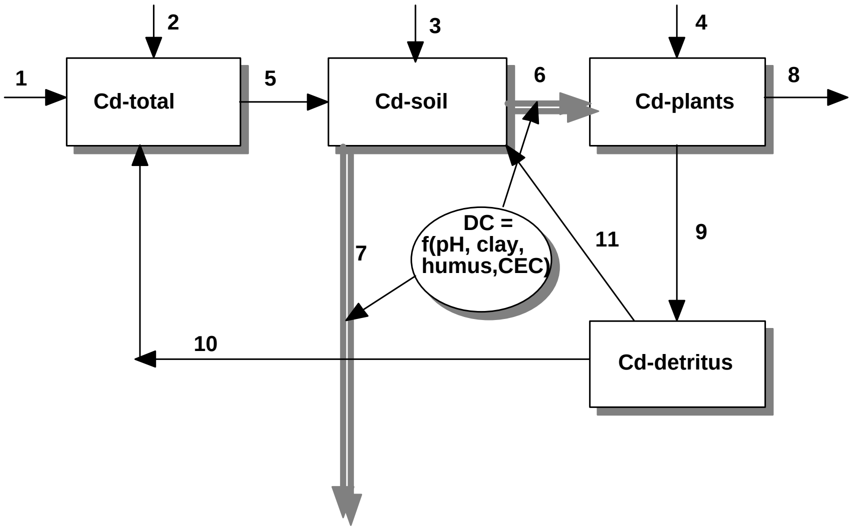

**FIGURE 4.** Conceptual diagram of the model. The model was made by use of the software STELLA. Boxes show state variables: cadmium total, cadmium soil, cadmium plants and cadmium detritus. The processes are (1) external sources of cadmium (fertilizers and sludge), (2)–(4) inputs of cadmium to the three compartments by air pollution, (5) transfer of cadmium from the slowly available cadmium pool to the more rapidly available cadmium pool, (6) uptake of cadmium by plants, (7) cadmium transported out of the root zone, (8) harvest, (9) cadmium in dead plant material, (10) mineralization of detritus cadmium to the slowly available cadmium pool, and (11) mineralization of cadmium detritus to the more rapidly available cadmium pool.

The transfer from Cd-total to Cd-soil indicates the slow release of cadmium, due to a slow decomposition of the more-or-less refractory material to which cadmium is bound. The cadmium uptake by plants is expressed as a first-order reaction, where the rate is dependent on the distribution coefficient, as only dissolved cadmium can be taken up. It is furthermore dependent on the plant species. As will be seen, the uptake is a step function that, for example here (grass), is 0.0005 during the growing season, and, of course, zero during the period after the harvest and until the next growing season starts. Cd-waste covers the transfer of plant residues to detritus after harvest. It is therefore a pulse function, which is 60% of the plant biomass here, as the remaining 40% has been harvested.

Cd-detritus covers a wide range of biodegradable matter, and the mineralization is therefore accounted for in the model by use of two mineralization processes: one to Cd-soil, and one to Cd-total. The first one is rapid and is given a higher rate for the first 180 days, as the addition of municipal sludge in this case is at day 0. The second one is at about the same rate, but as the cadmium is transferred to the Cd-total, the slow release rate is considered by the very slow transfer from Cd-total to Cd-soil.

Data from Jensen and Tjell[25] and Hansen and Tjell[24] were used for calibration and validation of the model. It was in this phase of the modeling procedure that it was revealed that three state variables for heavy metal in soil were needed to get acceptable results. It was particularly difficult to obtain the right values for heavy metal concentrations in the plants the second and third year after municipal sludge had been used as a soil conditioner. This use of models may be called experimental mathematics or modeling, where simulations with different models are used to deduce which model structure should be preferred. The results of experimental mathematics must, of course, be explained by examination of the processes involved, and here can be referred to the references given above.

The results of the validation phase are shown in Fig. 5 and Fig. 6, and as can be seen, the accordance between observations and model predictions is reasonably good.

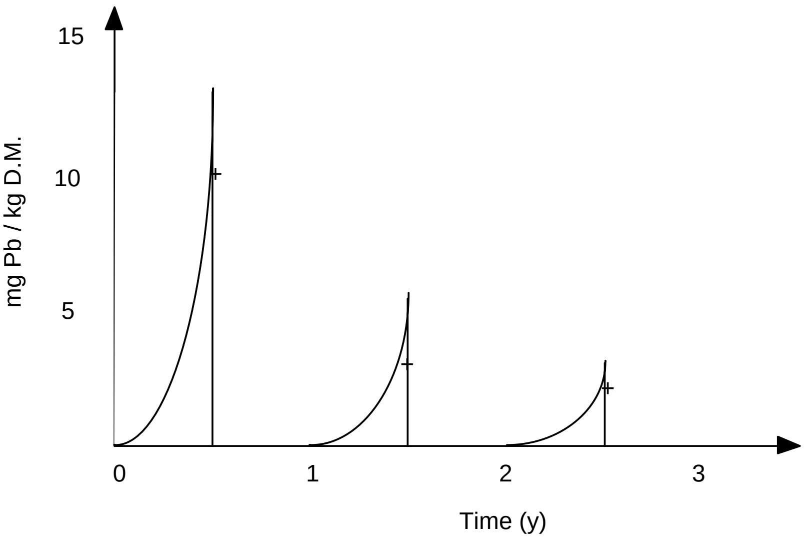

**FIGURE 5.** The model was validated by use of the lead concentration as a function of time (y) for salad plants. o gives the observations and the curve marked s gives the corresponding model predictions.

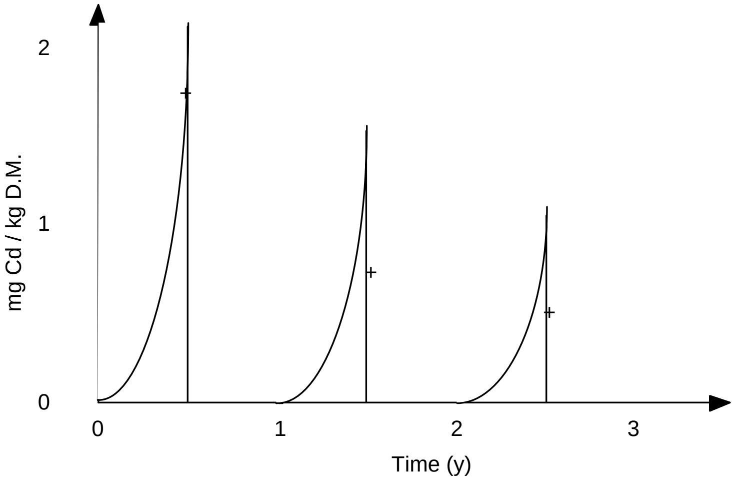

**FIGURE 6.** The model was validated by use of the cadmium concentration as a function of time (y) for red clover at third and fourth harvests. x gives the observations and the curve marked s gives the corresponding model predictions.

It is apparent from the validation that the developed model can explain the observations. A similar model may be used to examine how much heavy metal it is possible to remove per harvest, when the soil properties and the extent of contamination are known. A wider use of the model would, however, require that still more data from experiments with many plant species are used to test the model, including plant species that are especially effective at taking up heavy from contaminated soil.

It may be concluded from these results that the model structure must account for at least three state variables for the heavy metal in soil, to cover the ability of different soil components to bind the heavy metal by various processes.

The model has been validated on basis of 3 years' experiments and measurements, and it was clear from the model exercises that the atmospheric fallout and heavy metal in the plant residues were significant. Translocation of the heavy metal to various parts of the plant was not considered in the model, and this would be a natural next step to include in the model, as it is important to distinguish heavy metal concentrations in various parts of the plants, especially when removal of heavy metals by plants is used for phytoremediation.

The problem modeled is very complex, and many processes are involved. On the other hand, an ecotoxicological management model should be somewhat simple and not involve too many parameters. The model can obviously be improved, but it gives at least a first rough picture of the important factors determining the contamination of plants and the possibilities to use phytoremediation. For the most part, it is not possible to get very accurate results with toxic substance models, but on the other hand, as we want to use somewhat large safety factors, the need for high accuracy is not pressing.

## THREE OTHER POSSIBLE ECOHYDROLOGICAL MODELING CASES

This section gives three more examples of how ecological engineering could be used as a powerful tool in environmental management and how models could be used quantitatively in this context. Ecosystems with pulsing patterns often have greater biological activity and chemical cycling than systems with relatively constant patterns. A specific case, a study of an estuary in Brazil named the Cannaneia, will illustrate the recognition of pulsing forces and how it is possible to take advantage of them by using a model in an ecohydrological context. The shores of the islands in the estuary and the coast are very productive mangrove wetlands, and the entire estuary is an important nesting area for fish and shrimp. A channel was built to avoid flooding upstream, where productive agricultural land is situated. The construction of the channel has caused a conflict between farmers, who want the channel open, and fishermen, who want it closed, due to its reduction of the salinity in the estuary (the right salinity is of great importance for the mangrove wetlands). The estuary is exposed to tide, which is important for maintenance of a good water quality with a certain minimum of salinity. The conflict can be solved by use of an ecohydrological approach that takes advantage of the pulsing force (the tide). A sluice in the channel could be constructed to discharge the fresh water when it is most appropriate, which is determined by a model. The tide would, in this case, be used to transport the fresh water as rapidly as possible to the sea. The sluice should be operated by the model — closed when the tide is on its way into the estuary and open when the tide is on its way to the sea.

The eutrophication of the Mondego Estuary, Portugal, has increased during the last decades, due to discharge of nutrient-rich fresh water originating from agriculture. The estuary is exposed to freshwater discharge and to tidewater fluctuations. In parts of the estuary Enteromorpha spp. have replaced Zostera spp. In addition, the annual variation of Enteromorpha spp. is strongly dependent on the amount of fresh water, according to the following mechanisms: (1) a strong freshwater discharge will decrease salinity, and low salinity will inhibit macroalgae growth despite the increase in nitrogen in the water column and (2) dissolved nitrogen discharged from mainland with fresh water will increase N/P ratios, since phosphorus appears to be mostly released from sediment and becomes diluted in the water column. A model relating the growth conditions for Enteromorpha spp. and Zostera spp., determined by the salinity and the nutrient concentration, should be developed. By discharging the fresh water — a sluice is already constructed — at the time when Zostera spp. would have the best growth conditions (a combination of salinity and nutrient concentrations) and Enteromorpha spp. the worst conditions, it would be possible to utilize the ecohydrological principles to control, at least partially, the eutrophication of the estuary. A structurally dynamic model is under development for the application of this presented ecohydrological concept in ecosystem management

The internal lakes of the city of Copenhagen were modeled to examine the possibilities of reducing eutrophication. The lakes receive water from two sources: one with about 40 μg P/l

(Damhus Lake), and one with more than 100 µg P/l (Utterslev Mose). Unfortunately, there is not sufficient water of the low phosphorus concentration, because the model clearly shows that the problem of eutrophication can only be solved by reduction of the phosphorus input. However, the model has also been able to show that a shorter retention time and the utilization of the water with low phosphorus concentration from February to May would be able — without any costs — to reduce the eutrophication about 20%, measured by the chlorophyll a concentration at the spring bloom. It has furthermore been suggested to significantly reduce the phosphorus concentration from Utterslev Mose, which is the main source of water for the internal lakes of the city of Copenhagen, by use of wetlands. The wetlands should be constructed to treat the stormwater overflow in this district of Copenhagen and currently, the water from Utterslev Mose, to ensure that phosphorus concentration will be reduced permanently over the coming years. Nitrogen is removed easily by wetlands, due to denitrification, but phosphorus can only by reduced by harvest of the plants and by adsorption. In this case the wetland was constructed by use of a mixture of gravel and limestone with a particularly high adsorption capacity of phosphorus. The wetland is therefore able to remove phosphorus with an efficiency of 95%.

## DISCUSSION

The five cases described in this paper illustrate that it is possible, by a proper ecological management of the ecosystems, to produce a significant improvement in the environmental quality. This does not imply that environmental technology becomes redundant — on the contrary. Environmental and Ecological Engineering have to work hand in hand. They are both necessary. The experience has only shown that environmental technology is not sufficient, and that it must therefore be supplemented by other tools: cleaner technology, environmental legislation, and ecological engineering as discussed here.

In the case studies presented, it has been demonstrated how water quality can be improved by manipulating the hydrology of an ecosystem, and how phytoremediation can be used effectively to remove a sufficient amount of heavy metals from contaminated soil. Ecosystem management requires that we understand the processes of the ecosystem, in a holistic context, and the influence they have on the environmental quality. A proper management approach requires quantification of ecosystem behavior to the extent possible using ecological models. A holistic ecosystem management requires, furthermore, that the applied models reflect the key properties of the ecosystem. Recent developments have therefore been towards incorporating more and more ecosystem properties in the models to get better models — for instance individual-based models are now being used to track the differences between individuals in a population. One of the latest developments is structurally dynamic models, which account for adaptation and shifts in species composition. The experiences from the reservoir case presented here and from other model case studies show clearly that it is often of utmost importance to apply SDMs instead of the conventional biogeochemical dynamic models. This is probably often the case when ecological engineering methods are used, because they often involve radical changes in the prevailing conditions of the ecosystems.

Ecosystem management implies, furthermore, that the impact on the entire ecosystem is considered. This has been clearly emphasized in the latest environmental risk assessment of toxic substances, where not only the effects on organism-levels are considered, but also the effects on the entire ecosystem.

## CONCLUSIONS

The case studies presented here have clearly demonstrated that it is crucial to examine the possibilities of improving environmental quality by ecological engineering and modeling.

Holistic ecosystem management is clearly a useful supplementary tool to environmental technology.

The five cases furthermore illustrate that quantification of ecosystem behavior by an ecological model is a particularly effective approach to achieve results from this management strategy.
